# Virulence regulation in the fish pathogen *Pseudomonas plecoglossicid*a: an integrated overview

**DOI:** 10.3389/fcimb.2026.1928252

**Published:** 2026-09-07

**Authors:** Meiqin Mao, Qingpi Yan

**Affiliations:** Fisheries College, Jimei University, Xiamen, Fujian, China

**Keywords:** *Pseudomonas plecoglossicida*, secretion systems, temperature dependence, virulence factors, virulence regulatory network

## Abstract

The genomes of pathogenic bacteria typically encode a diverse array of virulence factors, which constitute the fundamental basis for resisting host immunity and establishing infection. Given the high energetic cost of virulence gene expression, pathogens must precisely orchestrate the activation and silencing of these genes in accordance with the infection process, thereby flexibly executing diverse biological functions within the constraints of their energy budget. To sustain efficient infection, pathogens have evolved multiple mechanisms governing gene expression, enabling them to maintain competitive advantages over both their hosts and microbial competitors across diverse niches. *Pseudomonas plecoglossicida* is an important Gram-negative pathogen in aquaculture that combines strong environmental adaptability with host invasiveness. It frequently causes explosive epidemics such as visceral white spot disease, inflicting substantial economic losses, and represents one of the principal pathogens threatening the sustainability of aquaculture. An in-depth dissection of its complex virulence mechanisms and hierarchical regulatory networks will provide critical molecular insights into the dynamic interplay between *P. plecoglossicida* and its hosts. Accordingly, this review focuses on the key virulence factors of *P. plecoglossicida*, including secretion systems (the type III secretion system and its effector PP_ExoU, and the fish-pathogen-specific type VI secretion system T6SS-1), extracellular products, the flagellar system, metal nutrient acquisition systems, and outer membrane barriers, together with their hierarchical regulatory networks comprising sigma factors, two-component systems, quorum sensing, the second messenger cyclic di-GMP, and small RNAs. We summarize the core functions of these virulence factors and dissect the molecular mechanisms by which this pathogen senses environmental signals and modulates virulence expression, aiming to provide a theoretical basis for the development of novel antivirulence strategies and therapeutic interventions.

## 1 Introduction

**Figure 1:**
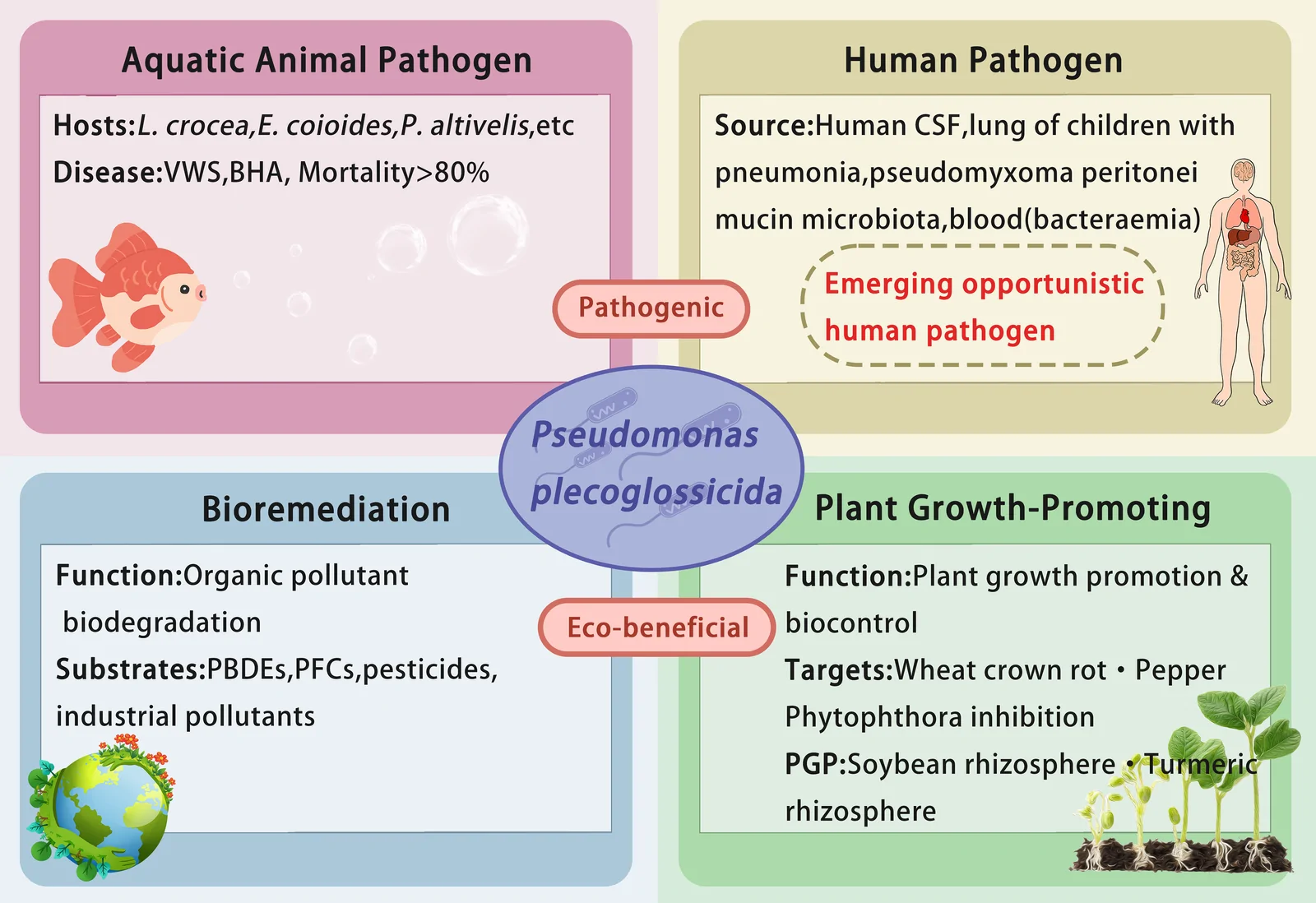
Schematic illustration of the dual pathogenic and eco-beneficial characteristics of *P. plecoglossicida*. As an aquatic animal pathogen, it causes visceral white spot disease (VWS) and bacterial hemorrhagic ascites (BHA) in economically important farmed fish, including *Larimichthys crocea*, *Epinephelus coioides*, and *P. altivelis*, with mortality rates exceeding 80% (upper left). It has also been identified as an emerging opportunistic human pathogen, recoverable from human cerebrospinal fluid (CSF), the lung of a child with pneumonia, the mucin microbiota of pseudomyxoma peritonei, and the blood of a bacteremia patient (upper right). Conversely, the species exhibits eco-beneficial traits, contributing to environmental bioremediation through the biodegradation of organic pollutants such as polybrominated diphenyl ethers (PBDEs), perfluorinated compounds (PFCs), and pesticides (lower left). Additionally, it functions as a plant growth-promoting bacterium (PGPB) colonizing the rhizospheres of soybean and turmeric, enhancing plant growth and providing biocontrol against wheat crown rot and *Phytophthora* blight in pepper (lower right).

The genus *Pseudomonas* occupies diverse ecological niches, including soil, water, and various plant and animal hosts (Silby et al., 2011; Tribelli and López, 2022; Khoshru et al., 2025). Its members encompass not only environmental strains with bioremediation capabilities, but also numerous pathogens that pose serious threats to plant and animal health (Silby et al., 2011; Khoshru et al., 2025). *Pseudomonas plecoglossicida* (*P. plecoglossicida*) is a representative aquatic animal pathogen within this genus, first isolated and identified by Nishimori et al. (2000) from lesions of bacterial hemorrhagic ascites (BHA) in diseased ayu (*Plecoglossus altivelis*) in Japan. Notably, remarkable phenotypic and genotypic divergence exists within *P. plecoglossicida* populations (**Figure 1**): some strains are highly virulent, causing high-mortality diseases in various farmed fish (Mao et al., 2024), whereas strains isolated from soil and natural water bodies resemble *Pseudomonas putida* in their physiological and biochemical properties and are non-pathogenic, instead exhibiting functions beneficial to the environment and agriculture (Raman et al., 2014; John et al., 2015). For example, strain NR_114226 acts as a plant growth-promoting rhizobacterium (PGPR) that effectively suppresses *Fusarium* crown rot of wheat and significantly promotes plant growth (Makhlouf et al., 2024). In addition, this bacterium has recently been detected in or isolated from human clinical settings, including the mucin microbiota of pseudomyxoma peritonei, as well as cases of pneumonia, meningitis, and bacteremia (Villarejo-Campos et al., 2023; Lin et al., 2024; Ahiakpa et al., 2025; Saegusa et al., 2026). The reported cases suggest two possible routes of human infection: intensive exposure to aquatic environments in immunocompetent individuals (Ahiakpa et al., 2025), and gut-derived opportunistic infection in immunocompromised hosts (Saegusa et al., 2026). Although there is currently no evidence of direct transmission from aquatic hosts to humans, its potential public health risk as an emerging opportunistic human pathogen has attracted increasing attention. This remarkable phenotypic and ecological functional diversity reflects the broad niche adaptability that *P. plecoglossicida* has developed through gene acquisition and functional specialization during adaptive evolution, and even points to its potential for cross-host infection.

*P. plecoglossicida* is a Gram-negative, aerobic, non-spore-forming, straight rod-shaped bacterium bearing polar flagella and exhibiting active swimming motility in liquid environments (Park et al., 2000; Li et al., 2020). Its host range covers a variety of economically valuable farmed fish, including ayu, large yellow croaker (*Larimichthys crocea*), orange-spotted grouper (*Epinephelus coioides*), hybrid pearl gentian grouper (*E. fuscoguttatus* ♀ × *E. lanceolatus* ♂), and rainbow trout (*Oncorhynchus mykiss*) (Park and Nakai, 2003; He et al., 2023c; Mao et al., 2024). Infection induces two typical diseases: bacterial hemorrhagic ascites, which mainly occurs in ayu, pejerrey (*Odontesthes bonariensis*), and rainbow trout (Liu et al., 2026); and visceral white spot disease (VWSD), which is common in large yellow croaker, orange-spotted grouper, and barramundi (*Lates calcarifer*), and is characterized by reduced feeding, decreased vitality, and white nodules of 1–2 mm in target organs including the spleen, kidney, and liver (Sun et al., 2020; Zhao et al., 2023; Mao et al., 2024). Epidemiological studies have shown that infection with this bacterium can cause mortality rates reaching 80–100%, resulting in severe economic losses to the aquaculture industry (Peng et al., 2026). As an important aquaculture pathogen, its outbreaks exhibit marked seasonality, occurring predominantly in spring and autumn when water temperatures range from 15 to 20 °C (Huang et al., 2019a). Such a complex ecological niche not only demonstrates the broad adaptability of this bacterium in both the environment and its hosts, but also determines its reliance on multi-layered regulatory networks for precise spatiotemporal control of virulence output, thereby enabling successful colonization and pathogenesis in different hosts.

Pathogenicity is essentially the outcome of the dynamic interplay between the pathogen’s capacity to resist host defense mechanisms and the host’s susceptibility to bacterial virulence factors (Zhou et al., 2023b; Soni et al., 2024), manifested as a continuous cascade in which the pathogen migrates to infection sites, adheres and colonizes, subsequently invades the host, suppresses or evades host defenses, acquires essential nutrients, and ultimately establishes systemic infection (Zhou et al., 2023b). The pathogenic success of *P. plecoglossicida* is attributable to its rich repertoire of virulence factors (**Table 1**), which precisely drives this process: the flagellar and chemotaxis systems (e.g., FliC) mediate directed migration to susceptible sites (He et al., 2025a; Peng et al., 2025); adhesive structures, biofilms, and outer membrane integrity factors (e.g., the LPS biosynthesis enzyme GalU) ensure stable colonization (Shu et al., 2023); the T3SS effector protein PP_ExoU disrupts host cell membranes to undermine immune defense (Zhang et al., 2017b); T6SS-1 and extracellular hydrolases mediate tissue invasion and granuloma formation (Tao et al., 2020); and iron/zinc acquisition systems (e.g., ZnuABC and FusA) secure essential elements in the nutrient-limited host environment (He et al., 2021; Huang et al., 2021; He et al., 2022). Notably, pathogen-host interaction is not unidirectional: bacteria must sense microenvironmental signals to dynamically adjust their virulence strategies, while host immunity exerts sustained selective pressure; therefore, virulence factor expression is not constitutive but relies on strictly coordinated multi-layered regulatory networks to achieve precise spatiotemporal control.

**Table 1:** Major virulence factors of *P. plecoglossicida* and their functional characteristics.

Category	Key representative	Primary function	Reference
Secretion systems	T3SS-ExsA	Master transcriptional activator of all T3SS genes; deletion increases LD_50_ ~190-fold, complete clearance from macrophages within 12 h	(Zhang et al., 2017a)
	T3SS-PopB/PopD	Insert into host membranes to assemble translocation pores; deletion blocks effector secretion, reduces flagellation, and drops adhesion rate from 96.2% to 9.6%, revealing cross-regulation with flagellar assembly	(Pan et al., 2023)
	T3SS-PP_ExoU	Ubiquitin-dependent phospholipase A1 activity; binds host cell membranes to induce membrane damage and mediates intramacrophage survival	(Zhang et al., 2017b)
	T6SS-1	Unique to fish-pathogenic strains, acquired via horizontal gene transfer; assembles a contractile injection apparatus, upregulated at low temperatures, driving pathogenesis and granuloma formation; synergizes with ester hydrolase for virulence, candidate for live attenuated vaccine	(Tao et al., 2020; Li et al., 2022a; Peng et al., 2026; Zhang et al., 2026)
	T6SS-2	Widely conserved, highest expression at optimal temperature; mediates interspecies antibacterial competition via effectors that degrade target bacterial nucleic acids, paired with immunity proteins to prevent self-intoxication	(Tao et al., 2020; Li et al., 2022b)
	T6SS-3	Residual incomplete gene clusters in environmental strains, downregulated at low temperature; no significant virulence or antibacterial phenotypes, suggesting a degenerating redundant system	(Tao et al., 2020; Li et al., 2022a, 2022b)
Extracellular products	Proteases, amylases, lipases, phosphatases	Degrade host extracellular matrix and membrane phospholipids to disrupt tissue barriers; enzymatic activities increase with temperature, significantly higher at 28 °C than at 12 °C and 18 °C	(Zhou et al., 2015)
	Cysteine proteases, aminopeptidases	Activity largely temperature-independent; functional roles remain undefined	
	Hemolysin	Weak hemolytic activity and temperature-insensitive, distinct from other fish pathogens that rely on hemolysins as core virulence determinants	
Motility and adhesion apparatus	FlgC, FlgK, FlgM	Basal rod structural protein (FlgC), hook-filament junction protein (FlgK), anti-sigma factor (FlgM); deletion significantly impairs chemotaxis, adhesion, and biofilm formation, reducing mortality by 55%–80%	(Sun et al., 2019a; Yuan et al., 2022b; Yang et al., 2023)
	FliA	Sigma-28 transcription factor, master regulator of the polar flagellar gene cascade; deletion downregulates 18 flagellar genes, attenuating motility, intracellular survival, and escape, reducing mortality by 30%	(Sun et al., 2019b)
	FliC	Flagellar filament protein; activates NF-κB pro-inflammatory pathway via Tlr5 and hijacks host glucose metabolism; deletion reduces virulence by 30%, significantly alleviating hepatic glycogen depletion and mitochondrial damage	(Peng et al., 2025)
	FliG, FliP, FliL	Basal body C-ring/MS-ring component (FliG), outer membrane export channel (FliP), flagellar assembly scaffold (FliL); deletion impairs motility and adhesion, increasing LD_50_ 2.1–11.5-fold	(Jiao et al., 2021; He et al., 2023b, 2023c; Shi et al., 2023, 2024)
	FliS	Flagellar filament chaperone; assists FliC secretion and folding across the membrane; deletion reduces flagellar assembly efficiency and motility	(Wu et al., 2022)
	LafK	σ^54^dependent transcriptional activator, master regulator of lateral flagellar genes; deletion reduces swarming motility by 50% and impairs biofilm formation, chemotaxis, and adhesion, reducing mortality by 30%	(He et al., 2025a)
Metal nutrient acquisition systems	TonB-ExbB-ExbD	Energizes outer membrane iron transport via proton motive force; deletion of TonB or ExbB globally impairs motility, chemotaxis, adhesion, and biofilm formation	(Hu et al., 2021; Tang et al., 2022)
	PvdE	ABC transporter mediating siderophore pyoverdine secretion; silencing results in non-lethal infection, significantly altering host Th1/Th2/Th17 differentiation and multiple immune pathways	(Xin et al., 2020)
	FusA	TonB-dependent ferredoxin receptor, upregulated under iron limitation; deletion impairs growth, adhesion, and biofilm capacity, involved in environmental adaptation	(He et al., 2022)
	ZnuABC	High-affinity zinc uptake system; ZnuA and ZnuC significantly upregulated during infection, essential for growth and intracellular survival under zinc limitation; transcription regulated by Fur	(He et al., 2021; Huang et al., 2021)
Outer membrane integrity factors	LPS-GalU	UDP-glucose pyrophosphorylase, involved in LPS and capsular polysaccharide biosynthesis; deletion abolishes O-polysaccharide side chains, thins cell wall, drastically reduces serum resistance, and impairs adhesion, invasion, and intracellular survival	(Shu et al., 2023)
	Mla pathway-MlaE	ABC transporter permease maintaining outer membrane lipid asymmetry; deletion increases sensitivity to polymyxin B and bile acids, attenuating virulence by ~30%, candidate for live attenuated vaccine	(Shu et al., 2025)

In this review, we focus on the virulence factors of *P. plecoglossicida* and their multi-layered regulatory networks, aiming to provide a systematic theoretical reference for elucidating its pathogenic mechanisms and developing novel antivirulence control strategies.

## 2 Virulence factors

The genome of *P. plecoglossicida* encodes a diverse repertoire of virulence factors, encompassing secretion systems, extracellular products (ECPs), the flagellar system, metal nutrient acquisition systems, lipopolysaccharide (LPS), and outer membrane proteins (OMPs) (**Table 1**). Acting in synergy, these virulence factors collectively drive the entire infection process of the pathogen.

### 2.1 Secretion system

#### 2.1.1 Type III secretion system (T3SS)

**Figure 2:**
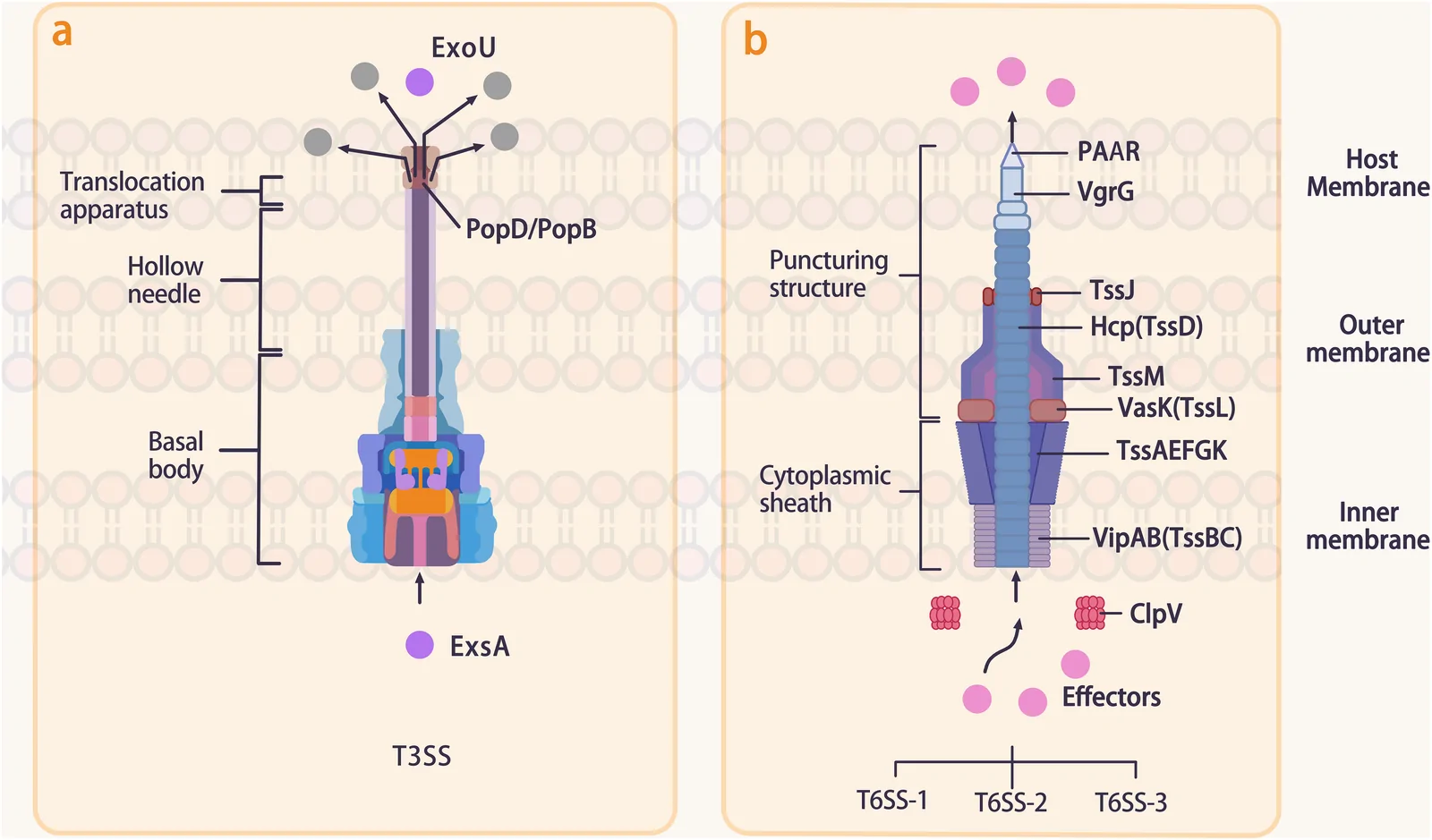
Structural architecture of the T3SS and T6SS in Gram-negative bacteria (Qin et al., 2022). **(a)** The T3SS injectisome is composed of a basal body, a hollow needle, and a translocation apparatus, which collectively form a syringe-like conduit that directly delivers effector proteins (e.g., ExoU) into the host cell cytoplasm; the AraC-type transcriptional activator ExsA serves as the master regulator governing T3SS gene expression. **(b)** The T6SS is a contractile nanomachine comprising a membrane-anchored complex (TssJ, TssM, VasK/TssL, TssAEFGK), a cytoplasmic sheath formed by VipAB (TssBC) oligomers, a puncturing structure consisting of stacked Hcp (TssD) hexamers capped with a VgrG–PAAR spike, and the ClpV that drives sheath disassembly and recycling. This apparatus propels effectors across both the inner and outer membranes of the bacterial envelope into recipient target cells. *Pseudomonas plecoglossicida* encodes three distinct T6SSs (T6SS-1, T6SS-2, and T6SS-3), each specialized for distinct biological functions.

Secretion systems are sophisticated protein-translocation machineries that mediate complex interactions between microorganisms and their environment as well as other organisms, constituting the core molecular basis for pathogens to establish virulence (Costa et al., 2015). The T3SS is a highly conserved protein-transport nanomachine in Gram-negative bacteria (Deng et al., 2017). Its syringe-like, multi-subunit transmembrane complex spans the bacterial inner and outer membranes and extends to the host cell membrane, injecting effector proteins directly into the host cytoplasm and thereby helping bacteria evade the host immune system (**Figure 2a**) (Deng et al., 2017). *P. plecoglossicida* harbors a 24-kb T3SS gene cluster located on a genomic island, whose gene organization and structural composition are highly homologous to those of *Pseudomonas aeruginosa* (Zhang et al., 2017a, 2017b). The functional architecture of this system comprises the needle complex, translocation apparatus, regulatory proteins, effector proteins, and chaperones, which act in concert to accomplish the transmembrane delivery of effectors.

The translocators PopB and PopD insert into the host membrane lipid bilayer and assemble into functional translocation pores, and maturation of the pore conformation provides the essential physical channel through which effector proteins such as PP_ExoU enter the host cytoplasm directly. Deletion of *popBD* not only blocks the transmembrane delivery of PP_ExoU but is also associated with defective flagellar assembly and reduced motility, ultimately leading to an overall attenuation of virulence (Pan et al., 2023). PP_ExoU is currently the only T3SS effector protein identified in *P. plecoglossicida*, and its features exemplify the combination of a conserved secretion machinery with functionally diverged effectors (Housley et al., 2011; Rahman et al., 2013; Zhang et al., 2017b). Like other ExoU-like proteins, it belongs to the patatin-like phospholipase family, requires eukaryotic-derived ubiquitin for activation, and targets the host cell membrane via its C-terminal membrane localization domain (MLD) and conserved arginine residues (Rahman et al., 2013; Tyson et al., 2015; Zhang et al., 2017b). However, PP_ExoU shares only 45% amino acid identity with ExoU from *P. aeruginosa*: the latter exerts PLA2 activity to trigger rapid and massive necrotic cell death and serves as a key virulence factor in acute infections (Shaver and Hauser, 2004), whereas PP_ExoU exhibits only PLA1 activity (with the catalytic dyad S132–D329) and causes mild, delayed cytotoxicity, suggesting a potential role in phagosomal escape and intracellular survival (Zhang et al., 2017b). Notably, the expression and secretion of PP_ExoU do not proceed constitutively but are strictly controlled by ExsA, the master regulator of the T3SS (Zhang et al., 2017b). In the Δ*exsA* mutant, PP_ExoU is neither expressed nor secreted, and T3SS function is completely silenced (Zhang et al., 2017b). ExsA thus acts as the master switch of the T3SS gene cluster, and the Δ*exsA* mutant exhibits a severe intracellular survival defect in macrophages, indicating that ExsA-mediated T3SS activation is decisive for *P. plecoglossicida* in evading host macrophage killing and establishing an intracellular replicative niche (Zhang et al., 2017a). Notably, the virulence of the Δ*exsA* mutant was not completely abolished, suggesting that *P. plecoglossicida* possesses additional T3SS-independent virulence factors and pathogenic pathways (Zhang et al., 2017a).

#### 2.1.2 Type VI secretion system (T6SS)

Another key mechanism playing an important role in sustaining infection is the T6SS (Wohlfarth et al., 2026). The T6SS is structurally homologous to the contractile bacteriophage tail and employs a “molecular spear” ejection mechanism driven by rapid sheath contraction, typically serving to inject toxic effector proteins into prokaryotic and eukaryotic cells (Wohlfarth et al., 2026). This system is assembled from three major complexes, namely the membrane complex (TssJ, VasK), the phage-like baseplate together with the inner tube and spike (TssA, Hcp, VgrG, PAAR), and the contractile sheath (VipA, VipB), and relies on ClpV for disassembly and component recycling, with each module fulfilling specific roles in membrane-anchored assembly, effector delivery, contractile firing, and cyclic regeneration, respectively (**Figure 2b**) (Jin et al., 2021).

Analysis by Navarro-Monserrat and Taylor (2023) revealed that over 50% of the 122 *Pseudomonas* genomes examined harbor multiple T6SS gene clusters, and multi-cluster pseudomonads display a stable pattern of functional division among these systems (**Table 2**). *P. plecoglossicida* encodes three T6SS subtypes, namely T6SS-1, T6SS-2, and T6SS-3, but the three clusters differ markedly in their organizational structure and evolutionary origins (Tao et al., 2020). Among them, T6SS-1 is found exclusively in fish-derived pathogenic strains and clusters within the same phylogenetic branch as virulence-associated T6SSs of *Bordetella petrii* and *Burkholderia* spp., suggesting that it was independently acquired through horizontal gene transfer (HGT) (Zhang et al., 2014; Luo et al., 2019a). *In vivo* infection experiments have further confirmed that T6SS-1 is the core virulence determinant driving the pathogenic phenotype: mutants lacking T6SS-1 or its core components ClpV and Hcp not only show drastic attenuation of virulence but also completely lose the ability to induce splenic granuloma formation, indicating that the contraction, firing, and recycling mechanical cycle and the effector delivery conduit are essential functional units for granuloma pathogenesis (Luo et al., 2019a; Jin et al., 2021; Zeng et al., 2022). In the exploration of live attenuated vaccines, the T6SS-1 *hcp* deletion mutant Δ*tssD*-1 proved safe for large yellow croaker, induced specific serum IgM and upregulated CD8α and MHCIα expression upon immunization, and conferred an RPS of 86.3% (Ye et al., 2021); likewise, the T6SS-1 cluster deletion mutant ΔS1Δ4885 was completely cleared by the host within 10 days post-infection, also demonstrating potential as a live attenuated vaccine candidate (Li et al., 2022a).

**Table 2:** Functional specialization of type VI secretion system (T6SS) clusters in *Pseudomonas* spp.

Species and strain	Cluster	Findings	References
*P. plecoglossicida* XSDHY-P/NZBD9/NB2011	T6SS-1	Essential virulence component	(Tao et al., 2020; Ye et al., 2021; Peng et al., 2026)
	T6SS-2	Interbacterial competition	(Tao et al., 2020; Jin et al., 2021; Li et al., 2022b)
	T6SS-3	No functional evidence	(Tao et al., 2020; Jin et al., 2021)
*P. aeruginosa* PAO1	H1-T6SS	Interbacterial competition	(Hood et al., 2010; Basler et al., 2013; Allsopp et al., 2017)
	H2-T6SS	Host interaction and metal nutrient acquisition	(Sana et al., 2012, 2015; Han et al., 2019; Wang et al., 2021a)
	H3-T6SS	Interbacterial competition and metal nutrient acquisition	(Russell et al., 2013; Lin et al., 2017)
*P. putida* KT2440/KT2442	K1-T6SS	Interbacterial competition	(Bernal et al., 2017, 2023; Nie et al., 2022)
	K2/3-T6SS	Conditional interbacterial competition	(Vázquez-Arias et al., 2025)
*P. fluorescens* F113	F1/F3-T6SS	Interbacterial competition and rhizosphere colonization	(Durán et al., 2021)
	F2-T6SS	Not expressed; function unknown	(Durán et al., 2021)
*P. syringae* DC3000	HSI-II	Intermicrobial competition (including anti-yeast activity)	(Haapalainen et al., 2012; Chien et al., 2020)
	HSI-I	No functional evidence	(Chien et al., 2020)
*P. protegens* Pf-5	Single core cluster	Interbacterial competition	(Whitney et al., 2013; Tang et al., 2018)

Progress has also been made in recent years at the effector level of the *P. plecoglossicida* T6SS-1 system. *paar*-1, an effector gene encoded within the T6SS-1 cluster, is regulated by T6SS-1 and its product is secreted in a T6SS-1-dependent manner; its deletion reduces biofilm formation, adhesion, antioxidant capacity, and Hcp-1 secretion, impairs intracellular survival in macrophages, and significantly decreases host mortality, tissue colonization, and splenic nodule formation, primarily through interference with the host Toll-like receptor signaling pathway (Zhang et al., 2026). Similarly, VgrG1a, a spike component of the T6SS-1 secretion apparatus, is itself directly involved in pathogenesis (Peng et al., 2026): it triggers an inflammatory storm via the TLR2/NOD2 pathway and suppresses AMPK-mediated gluconeogenesis and fatty acid oxidation, thereby disrupting hepatic immune-metabolic homeostasis, and its deletion increases host survival by 40% (Peng et al., 2026); the pathogenic role of *vgrG* has been validated in multiple infection models (Yang et al., 2025; Peng et al., 2026). Collectively, these findings indicate that the virulence output of T6SS-1 is closely linked to the interference of its effector molecules with host immune and metabolic pathways.

In contrast, T6SS-2 and T6SS-3 follow distinct evolutionary trajectories and functional outputs. Both are widely conserved across environmental and pathogenic strains (with T6SS-3 persisting only as an incomplete gene cluster in environmental strains) and share high homology with orthologs in *P. putida*, suggesting inheritance from a common ancestor and primary roles in interspecies antibacterial competition and niche occupation (Li et al., 2015, 2022b). Of these, T6SS-2 serves as the central weapon for interbacterial competition; its antibacterial activity is mainly mediated by the DNase effectors Txe1 and Txe4, which are encoded by the *vgrG*-2 and *vgrG*-5 clusters, contain an Rhs core domain and a C-terminal nuclease domain, and kill target bacteria by degrading their DNA (Li et al., 2022b). Each functional effector is paired with a cognate immunity protein (Txi1, Txi2, or Txi4) to prevent self-intoxication, while loading of effectors onto VgrG requires the assistance of the EagR adaptor protein (Li et al., 2022b). Competition assays demonstrated that Txe1 and Txe4 are the predominant antibacterial effectors (Li et al., 2022b). Notably, T6SS-2 contributes little to host pathogenicity; instead, it functions preferentially to eliminate commensal bacteria in the environment or the host gut, thereby reducing colonization resistance and clearing niche barriers for systemic infection (Tao et al., 2020). T6SS-3 exhibits no significant phenotype in either virulence or antibacterial activity, suggesting that it may represent a degenerating redundant system or one activated only under specific environmental stresses (Tao et al., 2020). This hierarchical division of labor among the three T6SSs enhances the dual adaptability of *P. plecoglossicida* to both the aquatic microecology and the host immune environment, rendering the T6SS a promising target for novel anti-infective therapies.

### 2.2 ECPs

ECPs are metabolic products secreted by bacteria during growth and are closely associated with the pathogenicity of aquatic pathogens (Li et al., 2025). The pathogenic components mainly include various enzymes and toxins, such as proteases, lipases, DNases, chitinases, and hemolysins (Sahu et al., 2011; Fan et al., 2024). These components are essential for bacterial adaptation, nutrient acquisition, intercellular signaling via membrane receptors, and bacterium-host interactions (Miyoshi, 2013; Martínez et al., 2026). In host-pathogen interactions, ECPs not only act as direct virulence factors (Sahu et al., 2011; MacKinnon et al., 2019; Yuan et al., 2022a; Song et al., 2025) but also exhibit immunogenicity, stimulating the host immune system to respond to infection (Yuan et al., 2022a; Li et al., 2025; Martínez et al., 2026). This dual role has been demonstrated in a variety of aquatic pathogens: ECPs from virulent strains of *Edwardsiella tarda* showed significantly stronger chemotactic activity toward host macrophages than those from attenuated strains (Wiedenmayer et al., 2008), and ECP preparations of *Aeromonas veronii* and *Aeromonas caviae*, administered by intraperitoneal injection, conferred RPS values of 75% and 65% in koi and crucian carp, respectively, both higher than those of the corresponding whole-cell inactivated vaccine groups (Song et al., 2018; Yuan et al., 2022a), suggesting that ECPs can serve as a candidate antigen source for aquatic vaccines. Different species produce different types of ECPs, and as bioactive substances, the magnitude of their activity directly determines the pathogenic capacity of the bacterium. Amylase is a case in point: reduced or abolished amylase activity directly leads to decreased bacterial virulence or impaired macrophage-killing ability (Best et al., 2018; Lin et al., 2021; Xu et al., 2024).

In addition, temperature plays a critical regulatory role in the activity output of ECPs. In *Aeromonas hydrophila*, temperature can reshape the direction of virulence functional output by selectively modulating the synthesis abundance of different ECP effector molecules (Sahu et al., 2011). In the mesophilic *Aeromonas salmonicida* strain SRW-OG1, the aerolysin and hemolysin encoding genes *aerA* and *hlyA* are significantly upregulated with increasing temperature, and the hemolytic activity and virulence of ECPs from high-temperature cultures are enhanced in parallel (Chen et al., 2022). The ECPs of *P. plecoglossicida* likewise exhibit marked temperature dependence, yet their regulatory pattern displays more complex features. Zhou et al. (2015) found that, across the three temperatures of 12 °C, 18 °C, and 28 °C, the activities of amylase, serine protease, aspartic protease, pepsin-like protease, chymotrypsin-like protease, and lecithinase in ECPs showed an overall increasing trend with rising reaction temperature, with enzymatic activities at 28 °C (a non-pathogenic temperature) significantly higher than those at 18 °C (the pathogenic temperature) and 12 °C (a non-pathogenic temperature). Moreover, the hemolytic activity of *P. plecoglossicida* ECPs is weak and temperature-insensitive (Zhou et al., 2015), in contrast to other pathogens that employ hemolysins as virulence-associated factors (Chen et al., 2022). However, the underlying mechanism of this “enzyme activity versus pathogenic temperature” disconnect in *P. plecoglossicida* remains to be elucidated. Accordingly, we hypothesize that these hydrolases may primarily serve environmental nutrient acquisition rather than virulence execution, and that during early colonization, excessive tissue-destructive enzymatic activity and the resulting acute necrosis would activate host immune alarm, conflicting with the chronic infection strategy of this bacterium, which is characterized by intracellular survival and granuloma formation (Smith et al., 2006; Barquero-Calvo et al., 2007). This hypothesis awaits validation through ECP profiling under different culture temperatures and *in vivo* functional studies.

### 2.3 Flagellar system

Flagella are not only essential structures for bacterial motility, but also play critical roles in the early stages of infection by promoting colonization and adhesion (Anderson et al., 2010; Tang et al., 2019b). *P. plecoglossicida* possesses a dual flagellar system composed of polar and lateral flagella (He et al., 2025a). Typically, the polar flagellum is constitutively expressed in liquid environments, driving individual swimming motility (McCarter, 2004), whereas the lateral flagella are induced on viscous surfaces or in host tissue environments, mediating swarming motility and biofilm spreading; they remain highly expressed throughout infection and serve as key promoters of pathogenicity (McCarter, 2004). Evolutionarily, flagella are highly homologous to the T3SS (Carroll and Liu, 2020; Halte and Erhardt, 2021) and share multiple functions, including biofilm formation, effector protein secretion, and adhesion to host cells (Duan et al., 2013; Barketi-Klai et al., 2014). The flagellar system can be divided into three structural domains: the basal body embedded in the cell envelope, the hook serving as a flexible joint, and the extracellular filament acting as a propeller (Carroll and Liu, 2020; Halte and Erhardt, 2021). The basal body consists of the LP ring, the rod, the MS ring, and the C ring, in which the rod connects the MS ring to the hook, while the C ring functions as the rotor that drives rotation and recruits substrate proteins (Johnson et al., 2021). Deficiency of flagellar structural components represses the expression of other flagellar genes and prevents flagellar assembly (Duan et al., 2013; Katharios et al., 2019; Halte et al., 2024). For example, deletion of basal body genes such as *flgC*, *fliP*, *fliG*, and *fliL* in *P. plecoglossicida* results in marked structural abnormalities, impaired motility, and attenuated biofilm formation, accompanied by a 2.1- to 11.5-fold increase in LD_50_ and a 50%–80% improvement in host survival. In addition, flagella can promote pathogenicity by modulating host immunity (Ma et al., 2021). The flagellar filament protein FliC triggers a strong pro-inflammatory response through TLR5 (Acharya et al., 2019), and its deletion directly reduces virulence by 30% and significantly alleviates host tissue damage (Peng et al., 2025).

Moreover, the ordered expression of flagellar genes is tightly regulated in a hierarchical manner (Dasgupta et al., 2003; Mo et al., 2022). In *P. aeruginosa*, flagellar gene expression is controlled through a four-tiered transcriptional hierarchy (designated classes I to IV), which ensures the temporal order and energy economy of flagellar assembly (Dasgupta et al., 2003). The dual flagellar systems of *P. plecoglossicida* are governed by distinct σ factors: the polar flagellum is regulated by a negative feedback loop composed of the alternative sigma factor FliA (σ^28^) and the anti-sigma factor FlgM (Sun et al., 2019a, 2019b; He et al., 2025a). Silencing of *fliA* downregulates 18 flagellum-associated genes and significantly impairs bacterial motility, intracellular survival, and lethality toward the host (Sun et al., 2019b), whereas silencing of *flgM* reduces host mortality by 75% and markedly reshapes multiple host immune-related pathways (Sun et al., 2019a). The lateral flagella, in contrast, are controlled by the master regulator LafK, a σ^54^-dependent transcriptional activator that initiates the cascade transcription of lateral flagellar genes by binding to the upstream promoter regions of early lateral flagellar operons, such as *flhABfliRQPNM*, and recruiting the RNA polymerase holoenzyme (He et al., 2025a). He et al. (2025a) demonstrated that deletion of *lafK* significantly downregulates the *flhABfliRQPNM* gene cluster, attenuates swarming motility by nearly 50%, and concomitantly impairs biofilm formation, chemotaxis, and adhesion. In infection assays, the Δ*lafK* strain increased grouper survival by 30%, with markedly reduced tissue bacterial loads and pathological damage, while pro-inflammatory cytokines were significantly downregulated at later stages owing to accelerated bacterial clearance (He et al., 2025a). Whether cross-regulation exists between the LafK and FliA–FlgM systems in *P. plecoglossicida* remains unclear.

### 2.4 Metal acquisition

During host-pathogen interactions, beyond direct physical invasion and immune activation, there exists a “metabolic tug-of-war” over essential nutrients, known as nutritional immunity (Lonergan and Skaar, 2019). The host restricts pathogen growth and proliferation by sequestering key metal ions such as iron (Fe^3+^) and zinc (Zn^2+^), whereas pathogens have evolved efficient and specific uptake systems to breach this blockade (Lonergan and Skaar, 2019; Ni and Liu, 2025). To overcome this challenge, *P. plecoglossicida* has evolved multiple metal acquisition systems capable of efficiently scavenging metals from the extracellular environment.

#### 2.4.1 Iron acquisition system

**Figure 3:**
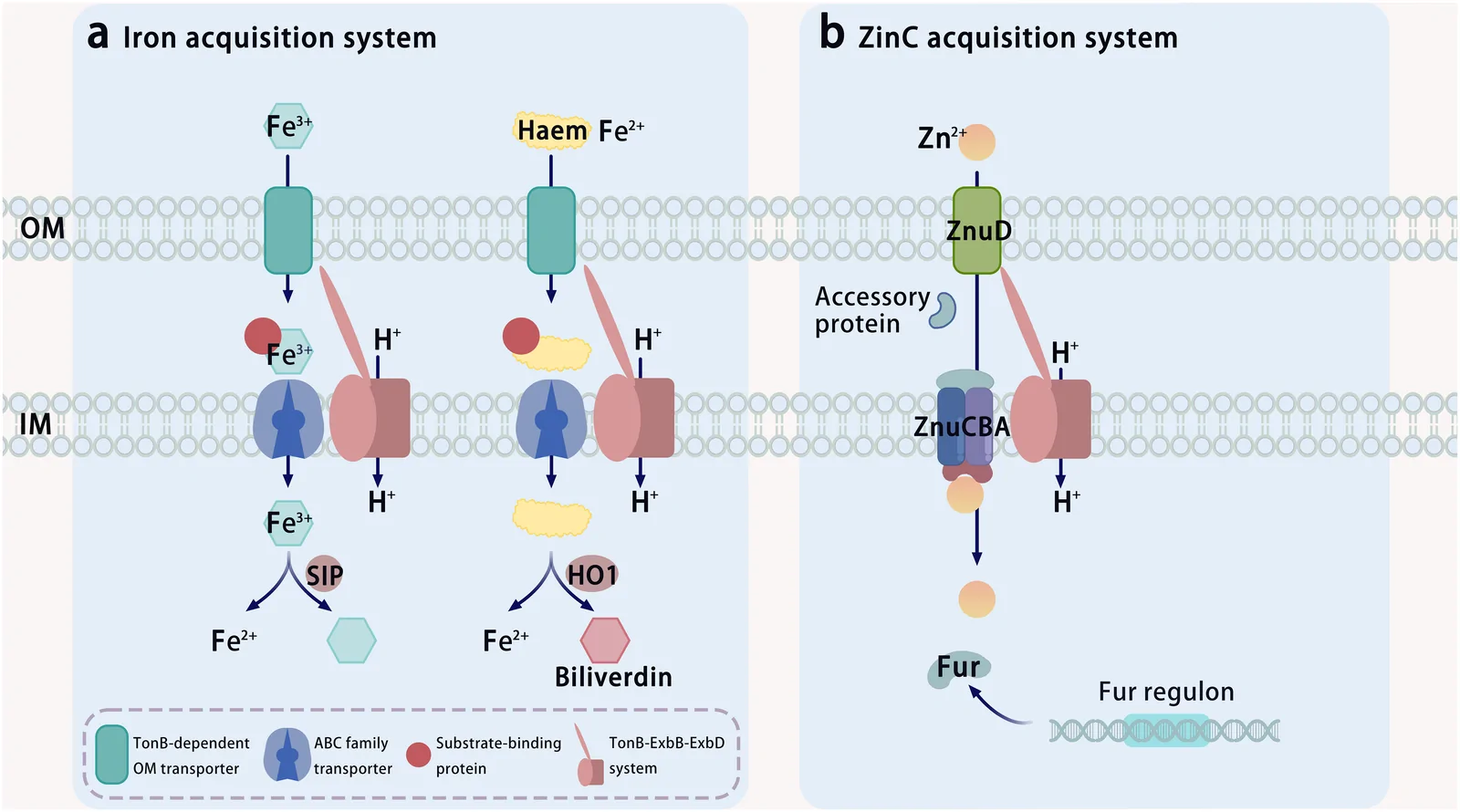
Iron and zinc acquisition systems in Gram-negative bacteria. **(a)** Iron acquisition system. Ferric iron (Fe^3+^) and heme-bound ferrous iron (Fe^2+^) are captured by distinct TonB-dependent outer membrane (OM) transporters. Translocation across the outer membrane is energized by the proton motive force (H^+^) provided by the TonB–ExbB–ExbD complex. Periplasmic substrate-binding proteins (SBPs) and ABC-family inner membrane (IM) transporters subsequently mediate transport across the inner membrane into the cytoplasm. Intracellular iron is liberated via bacterioferritin-associated ferredoxin (SIP)-mediated reduction or heme oxygenase (HO1)-catalyzed heme degradation, releasing free Fe^2+^. **(b)** Zinc acquisition system. Zn^2+^ is captured at the outer membrane by ZnuD (TonB-dependent transporter), assisted by an accessory protein, and delivered to the periplasm for subsequent import across the inner membrane by the ZnuCBA ABC transporter system. Intracellular Zn^2+^ concentrations are sensed by the Fur transcriptional regulator, which modulates expression of the Fur regulon to maintain zinc homeostasis.

Iron is the most widely used transition metal in biological systems (Lemos and Balado, 2020). To overcome host-imposed iron limitation, Gram-negative bacteria have generally evolved a highly regulated iron uptake system comprising multiple coordinated steps, consisting of three functionally complementary core modules: outer membrane receptors that specifically recognize iron sources, a transmembrane energy transduction system (the TonB-ExbB-ExbD complex) that provides the driving force, and inner membrane ABC transport systems responsible for cytoplasmic delivery (Gouda et al., 2023) (**Figure 3a**). The functional integrity of this system directly determines the survival and pathogenic capacity of pathogens in the iron-limited host environment.

Within the iron uptake system, outer membrane transport serves as the first rate-limiting checkpoint for iron entry into bacteria (Hussein et al., 2024). Large molecules such as siderophore-iron complexes or heme cannot diffuse through porins and must be actively translocated across the outer membrane via TonB-dependent transporters (TBDTs) (Hussein et al., 2024). Among these, FusA, a core TBDT iron uptake receptor, is significantly upregulated under iron starvation and at the pathogenic temperature (18 °C), and deletion of *fusA* impairs the adhesion, tissue dissemination, and colonization capabilities of *P. plecoglossicida* (He et al., 2022). The siderophore itself is secreted by an ABC transporter encoded by *pvdE*, which is responsible for the extracellular export of pyoverdine, the *Pseudomonas*-specific siderophore that chelates scarce free iron in the environment (Xin et al., 2020). The TonB-ExbB-ExbD system acts as a transmembrane energy-coupling complex that harnesses the proton motive force to drive outer membrane iron transport, and the genes encoding its key components, *tonB* and *exbB*, are significantly upregulated in infected spleens (Hu et al., 2021; Tang et al., 2022). These components influence pathogenicity by regulating motility, adhesion, and biofilm formation in *P. plecoglossicida* (Hu et al., 2021; Tang et al., 2022). After entering the periplasm via the TBDT-TonB system, siderophore-iron complexes are captured by specific periplasmic binding proteins and delivered to inner membrane ABC transporters to complete cytoplasmic delivery (Mosbahi et al., 2018).

#### 2.4.2 Zinc acquisition system

Zinc, the second most abundant transition metal after iron, serves as a structural, catalytic, and signaling component that is not only essential for the growth and development of organisms, but also participates in bacterial metabolism and the regulation of various virulence factors (Wang et al., 2021b). Upon host infection, *P. plecoglossicida* triggers nutritional immunity to restrict its proliferation *in vivo*, which is mainly manifested as decreased serum zinc, zinc accumulation in the spleen, and zinc compartmentalization within phagosomes (Botella et al., 2011; Sheldon and Skaar, 2019). To overcome zinc limitation, *P. plecoglossicida* has evolved a high-affinity uptake system centered on ZnuABC. This system consists of the periplasmic binding protein ZnuA, the inner membrane permease ZnuB, and the ATP hydrolase ZnuC, which efficiently pump extracellular free zinc into the cytoplasm (**Figure 3b**) (Ma et al., 2015). During infection, *znuA* and *znuC* are significantly upregulated, indicating that *P. plecoglossicida* actively responds to host-imposed zinc limitation signals (He et al., 2021; Huang et al., 2021). Reduced zinc uptake directly weakens the virulence output and host adaptability of *P. plecoglossicida* (He et al., 2021; Huang et al., 2021). Notably, *P. plecoglossicida* lacks the outer membrane transporter ZnuD and the regulator Zur. In bacteria possessing ZnuD, ZnuD transports extracellular Zn^2+^ across the outer membrane into the periplasm, whereupon the ZnuABC system completes the subsequent uptake (Calmettes et al., 2015; Qamsari et al., 2020). In the absence of *znuD* in *P. plecoglossicida*, how extracellular zinc crosses the outer membrane into the periplasm and is captured by the ZnuABC system remains unclear.

Beyond uptake systems, bacteria must also precisely control intracellular metal homeostasis. Transition metals are essential components of multiple metabolic pathways (Sarvan et al., 2018). However, excessive metals cause metal toxicity in bacteria, which is also exploited by the host as an antimicrobial strategy. To balance metal limitation against metal overload, bacteria have evolved metalloregulatory proteins for precise control (Chandrangsu et al., 2017). In most Gram-negative bacteria, expression of the ZnuABC system is tightly controlled by Zur (zinc uptake regulator), which represses the operon under zinc-replete conditions and dissociates to relieve repression upon zinc starvation (Bui and Inaba, 2024). The *P. plecoglossicida* genome, however, lacks a canonical Zur homolog and has not evolved an independent zinc-specific transcriptional regulatory layer (Huang et al., 2021). As compensation, its Fur can directly bind the promoter region of the *znuCBA* operon and relieve the repression of zinc uptake gene expression by sensing fluctuations in intracellular iron concentration; the resulting Fur-centered compensatory regulatory mechanism for zinc uptake provides a critical safeguard for *P. plecoglossicida* to maintain virulence in zinc-limited host environments (Huang et al., 2021). Similar iron-zinc cross-metal regulation has also been reported in *Neisseria meningitidis*, in which the zinc outer membrane receptor gene *znuD* is dually regulated by Fur and Zur (Kumar et al., 2012); the difference is that this species retains Zur, with Fur playing only an auxiliary role, whereas in *P. plecoglossicida* Fur independently assumes the regulation of zinc uptake in the absence of Zur, representing an extreme form of metalloregulatory plasticity within the Fur family.

### 2.5 Outer membrane barrier

The envelope of Gram-negative bacteria consists of two membranes, the inner and outer membranes (Henderson et al., 2016). The outer membrane (OM) is essentially an asymmetric lipid bilayer, with LPS predominating in the outer leaflet and phospholipids in the inner leaflet (Zhang et al., 2013). It is precisely this lipid asymmetry that constitutes the structural basis of the OM permeability barrier, enabling it to withstand external insults such as antibiotics (Tan and Chng, 2024). Maintenance of this asymmetry depends not only on the outward transport of LPS but also on the retrograde trafficking of phospholipids back to the inner membrane (Tan and Chng, 2024). The Mla pathway is the highly conserved system that executes this retrograde transport, comprising the outer membrane protein MlaA, the periplasmic protein MlaC, and the inner membrane transporter complex MlaBDEF (Kaur and Mingeot-Leclercq, 2024). Once the Mla pathway is compromised, phospholipids in the outer leaflet cannot be returned to the inner membrane and instead undergo phase separation with LPS to form phospholipid patches, leading to collapse of the OM barrier and a sharp increase in permeability, which in turn heightens bacterial susceptibility to environmental stresses (Malinverni and Silhavy, 2009; Guest et al., 2023). Notably, the role of the Mla system in niche adaptation and virulence regulation exhibits marked species specificity (Fernández-Calvet et al., 2018; Baarda et al., 2019; Nasu et al., 2022). In *P. plecoglossicida*, MlaE affects virulence by maintaining membrane asymmetry. The OM barrier disruption and virulence attenuation caused by *mlaE* deletion arise directly from structural damage to the outer membrane rather than from indirect regulation of T6SS-1 (Shu et al., 2025). A recent study by Ahat et al. (2026) further confirmed the feasibility of targeting the Mla system against *P. plecoglossicida*: the triple-gene deletion mutant Δ*mlaE*Δ*ompR*Δ*deoR* is highly attenuated and safe for large yellow croaker, and immunization via intraperitoneal injection and immersion conferred RPS values of 80.43% and 76.92%, respectively, accompanied by early upregulation of pro-inflammatory factors and a sustained increase in serum-specific IgM (Ahat et al., 2026).

LPS is composed of three parts: lipid A, core polysaccharide, and the O-antigen (Tang et al., 2025). Among them, the O-polysaccharide antigen, a long-chain polysaccharide protruding from the outermost bacterial surface (Mastroeni et al., 2026), not only confers the characteristic rough and undulating contour of the bacterial surface, but also serves, owing to the plasticity of its chain length and composition, as a key structure for evading host immune attack and adapting to complex environments (Liu et al., 2012). UDP-glucose is an activated sugar donor essential for the biosynthesis of LPS, capsular polysaccharides, and exopolysaccharides, and its production is catalyzed by UDP-glucose pyrophosphorylase encoded by *galU* (Zhou et al., 2021). Once *galU* is mutated, the UDP-glucose supply is interrupted and O-polysaccharide side-chain synthesis is blocked; consequently, the surface of *P. plecoglossicida* loses the spatial protrusions formed by long O-antigen chains and becomes smooth, while the thickness of the cell wall (the glycan-containing layer) is also significantly reduced (Shu et al., 2023). Although the specific molecular pathways by which O-polysaccharide deficiency reduces adaptability remain incompletely understood, the cascading effects of “deglycosylation” weaken the adaptability of *P. plecoglossicida* at multiple levels, including reduced serum resistance, decreased adhesion to host cells, and drastically attenuated virulence (Shu et al., 2023). Studies on *galU* in *P. plecoglossicida* and various other Gram-negative bacteria have consistently demonstrated that this gene is essential for maintaining LPS integrity, stress tolerance, and pathogenic capacity (Zhou et al., 2021; Shu et al., 2023; Tsai et al., 2025).

OMPs, anchored on the outer membrane surface, constitute the first point of contact through which the host immune system recognizes pathogens and, endowed with both structural functions and immunodominance, have long been regarded as ideal vaccine candidates. The outer membrane porin OprF is highly conserved among *Pseudomonas* species (He et al., 2024b; Wilkes et al., 2025). As a subunit vaccine, recombinant OprF not only induces a strong immune response in large yellow croaker, but also confers an RPS of up to 70.60% against *P. plecoglossicida* infection (He et al., 2024b). Building on this, the lipoprotein OprI represents another important component of the *P. plecoglossicida* outer membrane (Li et al., 2026). Li et al. (2026) developed a safe and effective recombinant adenovirus-vectored vaccine co-expressing OprF and OprI, which induces more comprehensive humoral and cellular immune responses via immersion immunization and increases the RPS of large yellow croaker against *P. plecoglossicida* to 79.07%. This leap in protective efficacy suggests that OprF and OprI are functionally complementary.

## 3 Virulence regulatory systems

In the dynamic interplay between bacteria and their hosts, a pathogen must respond rapidly and precisely to biochemical and physical changes in both the internal and external environments to successfully invade and survive within the host, which involves the remodeling of cellular programs at both the transcriptional and translational levels (Qiu et al., 2013). Recent studies have revealed that bacteria behave like a well-trained “symphony orchestra”, coordinating the expression and activity of diverse regulatory factors in response to multiple signals from the host or the environment. The “conductor” of this orchestra is the bacterial stress-response regulatory machinery, which senses and integrates key environmental cues such as temperature, osmolarity, and nutrient availability, thereby precisely orchestrating the pathogenic program through complex regulatory networks. Therefore, this section systematically elaborates how five classes of core regulatory elements, namely σ factors, two-component systems (TCSs), quorum sensing (QS), cyclic diguanylate (c-di-GMP), and non-coding small RNAs (sRNAs), regulate bacterial virulence gene expression during pathogen-host interactions.

### 3.1 σ factors

At the most fundamental level, σ factors determine where RNA polymerase initiates transcription by binding to specific promoter sequences, and multiple σ factors regulate gene expression in response to different environmental stimuli by recognizing distinct conserved promoter sequences (Nguyen et al., 2025). Bacteria typically possess one housekeeping σ factor and multiple alternative σ factors, with the former responsible for initiating transcription from the majority of promoters and the latter controlling the transcriptional initiation of specific subsets of genes (Qiu et al., 2013). The number and types of σ factors vary considerably among bacterial species (Mauri and Klumpp, 2014), generally reflecting their ecological niches and lifestyles.

Based on the sequence specificity of promoter recognition, the σ factors of *P. plecoglossicida* fall into two families: the σ^70^ family, which recognizes conserved hexanucleotide sequences at the -35 and -10 regions (the Pribnow box), and the σ^54^ family, which recognizes promoter sequences at the -24 and -12 regions (Staroń et al., 2009). The housekeeping σ factor RpoD, a member of the σ^70^ family, serves as the core σ subunit of bacterial RNA polymerase and is essential for sustaining fundamental cellular activities (Xin et al., 2022). Apart from RpoD, all other σ factors encoded by *P. plecoglossicida* are alternative σ factors, presenting a highly hierarchical regulatory architecture. FliA precisely controls the temporal sequence of flagellar biogenesis through the FlgM/FliA checkpoint (Sun et al., 2019a, 2019b); RpoE integrates outer membrane stress and temperature signals via a regulated intramembrane proteolysis (RIP) cascade, serving as an upstream hub that regulates T6SS expression, biofilm formation, and intracellular survival (Zhang et al., 2022); SigX, another extracytoplasmic function (ECF) σ factor, participates in bacterial pathogenicity and modulates host immune responses (Sun et al., 2018); and RpoN, together with its dependent transcriptional activators (such as RK21_RS10315), links flagellar assembly to fundamental physiological processes such as nitrogen metabolism (Zhang et al., 2019). By competing for the RNA polymerase core enzyme and engaging in cross-regulation, these σ factors form a complex transcriptional network that enables the bacterium to achieve precise spatiotemporal control of gene expression across diverse microenvironments inside and outside the host, constituting the core molecular basis for the emergence of its virulence. At present, a panoramic map of the σ factor regulatory network in *P. plecoglossicida* (particularly the complete target gene repertoire of the σ^54^ regulon and the signal-specificity mechanisms of ECF σ factors) remains to be systematically elucidated.

### 3.2 TCSs

**Figure 4:**
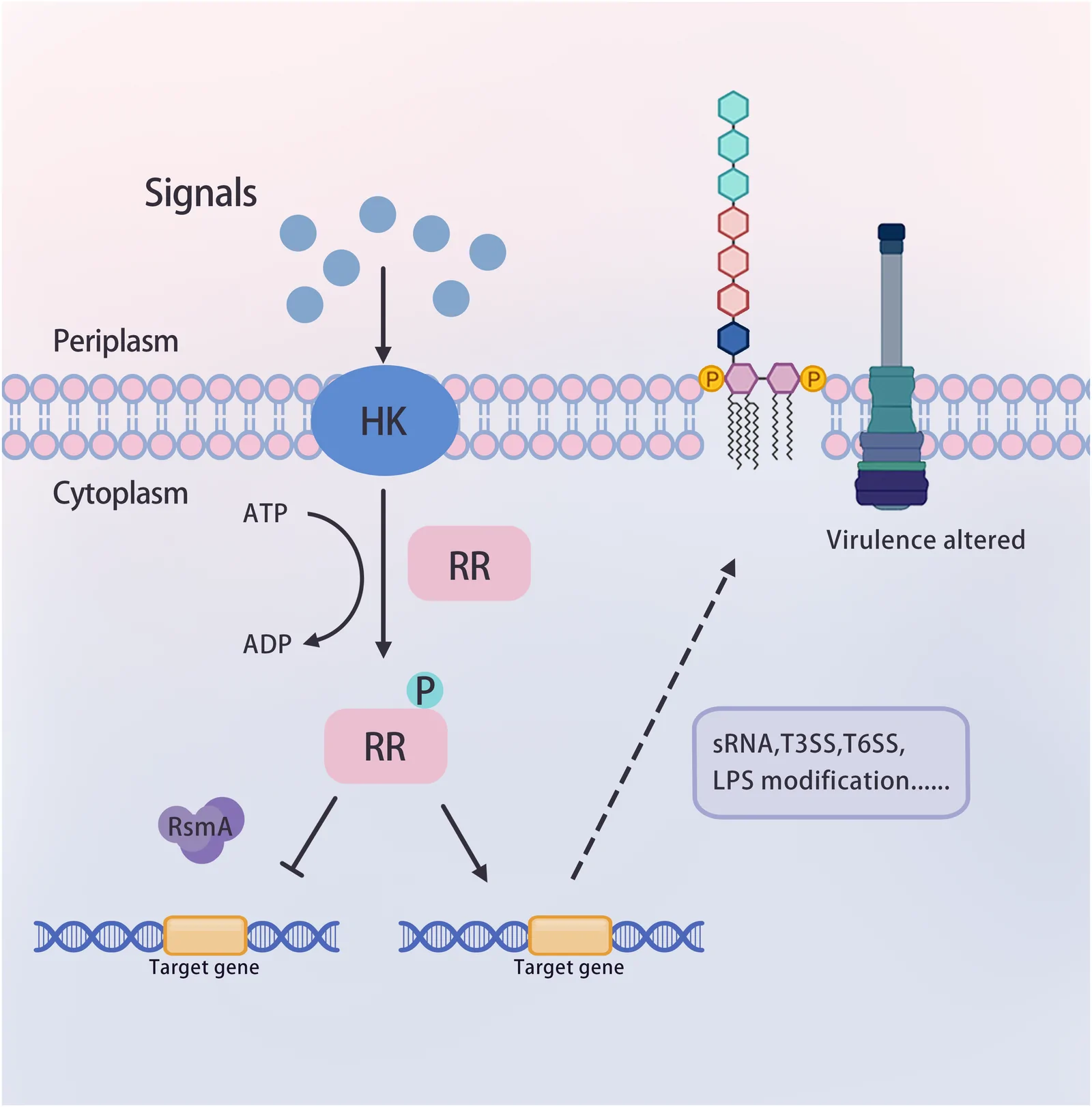
TCS mediated regulatory mechanism of bacterial virulence. Upon perceiving environmental cues, the membrane-anchored HK undergoes ATP-dependent autophosphorylation and transfers the phosphate group (P) to its cognate RR. The resulting phosphorylated RR (RR~P) functions as an activated transcription factor that selectively activates or represses downstream target gene expression. This signaling axis coordinately governs the expression of diverse virulence determinants, including sRNAs, T3SS, T6SS, and LPS phosphorylation/modification, ultimately shaping bacterial virulence phenotypes.

TCSs are the predominant regulatory form by which bacteria respond to environmental changes, consisting of a histidine kinase (HK) and a response regulator (RR) (Groisman et al., 2021; Mao et al., 2025a). Upon sensing environmental signals, the HK autophosphorylates and transfers the phosphoryl group to the RR (Groisman, 2016); the phosphorylated RR then regulates downstream gene expression by enhancing its affinity for DNA targets, playing a central role in bacterial survival, metabolism, and virulence (Groisman, 2016) (**Figure 4**). Among these systems, PvgAS senses the coordinated input of Na^+^ concentration (the dominant signal) and temperature (a secondary modulating factor) to positively drive the transcription of the T6SS-1 and T3SS gene clusters, with effects extending to genes involved in multiple metabolic pathways (Tao et al., 2024; Ding et al., 2025). Within this network, T6SS-1 serves as the core effector apparatus mediating PvgAS-dependent pathogenicity, and its effector protein secretion directly determines splenic granuloma formation and the outcome of acute infection (Tao et al., 2024; Ding et al., 2025). Thus, PvgAS acts as a dual ion-temperature signal switch that precisely modulates the virulence gene output of the bacterium.

GacS/GacA is a conserved global regulatory system in the genus *Pseudomonas*, whose core logic is to control the activity state of the RNA-binding protein RsmA through the Gac/Rsm signaling cascade (Gooderham and Hancock, 2009). GacA activates the transcription of the anti-RsmA small RNAs (RsmY/Z), which sequester the RsmA protein and thereby relieve its translational repression of mRNAs encoding flagellar, biofilm, and virulence factors (Gooderham and Hancock, 2009). In *P. plecoglossicida*, deletion of *gacS* results in marked downregulation of flagellar gene clusters, biofilm formation, and the T6SS-2 component genes (*tssB* and *tssC*), accompanied by significant upregulation of an RsmA-like gene. This finding is highly consistent with the classical model established in *P. aeruginosa*, with both pointing to the accumulation of free, active RsmA as the central cause of the pleiotropic repression of virulence genes following *gacS* deletion (Zhou et al., 2023a). In addition, the *gacS* mutant is impaired in invasion ability; a live attenuated vaccine constructed from this mutant is safe for large yellow croaker and confers RPS values of up to 71.05% (intraperitoneal injection) and 76.31% (immersion), representing a potential live attenuated vaccine candidate (Kang et al., 2024).

PhoP/PhoQ is the core TCS in *P. plecoglossicida* that senses the host microenvironment (low Mg^2+^, weak acidity, and cationic antimicrobial peptides (CAMPs)) (Mao et al., 2025b, 2025c). Upon signal perception, PhoQ activates PhoP through phosphorylation, and the latter globally regulates the expression of stress adaptation and virulence genes. Deletion of *phoQ* causes a series of physiological defects and increases susceptibility to polymyxin B (Mao et al., 2025b, 2025c). Meanwhile, the mutant exhibits markedly reduced virulence following grouper infection, with key host immune genes displaying attenuated kinetic patterns, indicating that the PhoP/PhoQ system indirectly “calibrates” the magnitude of the host immune response by positively regulating bacterial virulence output, thereby serving as a central regulatory node for the adaptation of this bacterium to the host microenvironment (Mao et al., 2025b, 2025c).

EnvZ/OmpR is a classical TCS that senses environmental osmolarity; it belongs to the OmpR family together with PhoP/PhoQ, although the two systems differ in their sensing duties (Xiao et al., 2022). This system regulates the expression of the outer membrane porins OmpF and OmpC in response to osmolarity changes in the growth medium (Kenney and Anand, 2020). In addition, EnvZ/OmpR plays a key role in the pathogenesis of various bacterial species (Du et al., 2022; Xiao et al., 2022). In *P. plecoglossicida*, deletion of *ompR* directly weakens bacterial virulence output and the capacity for survival and colonization (He et al., 2023a). Given the synergistic regulatory interplay between EnvZ/OmpR and RpoS, the Δ*ompR*Δ*rpoS* double mutant exhibits an approximately 10^7^-fold reduction in virulence compared with the wild-type strain, and the corresponding live attenuated vaccine confers RPS values of 72.25% (challenge at 4 weeks post-vaccination) and 64.90% (challenge at 6 weeks post-vaccination), and is regarded as a promising live attenuated vaccine candidate against *P. plecoglossicida* infection (He et al., 2023a).

### 3.3 QS systems

**Figure 5:**
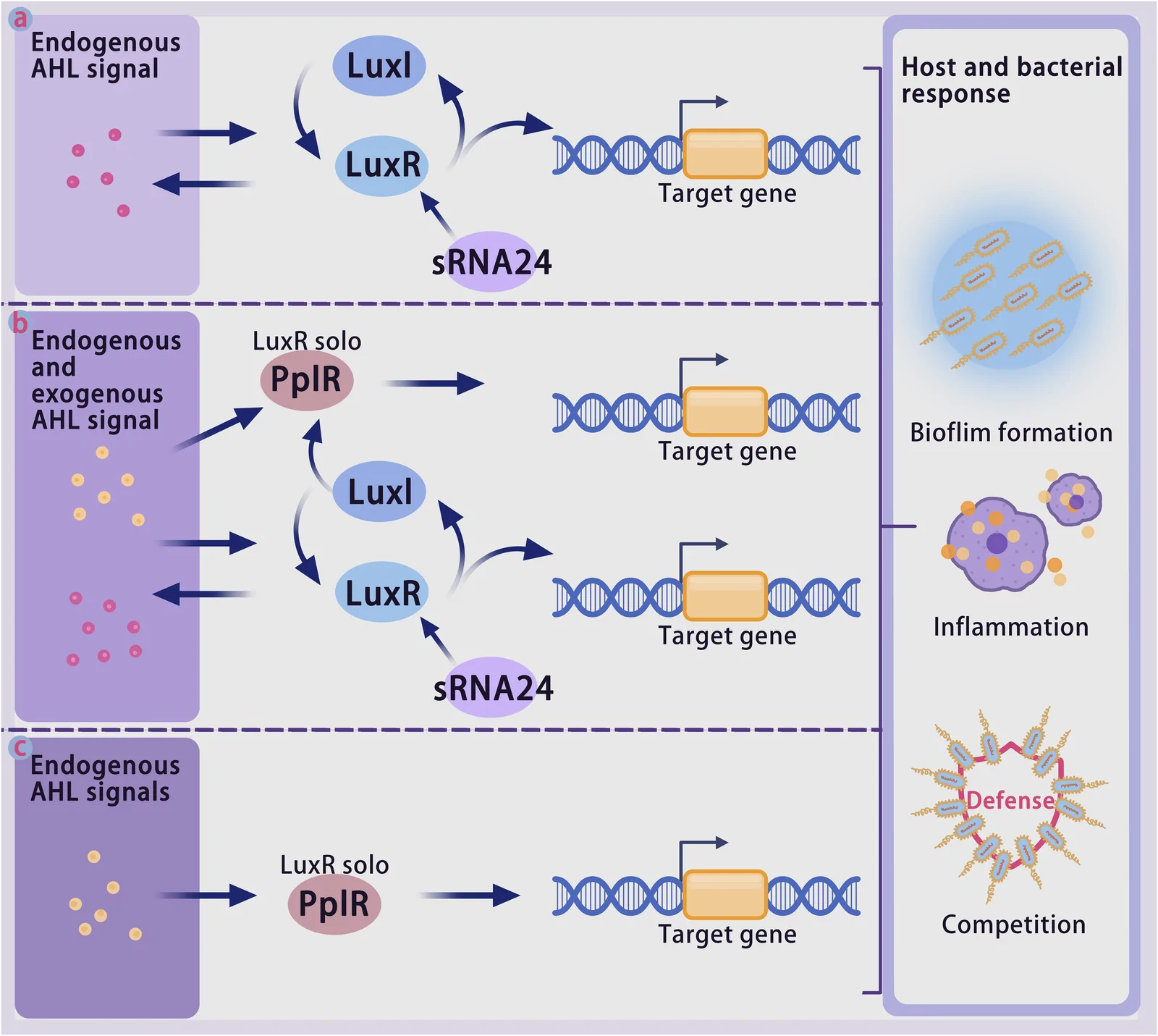
Regulatory mechanisms of the AHL-mediated QS system. **(a)** Canonical AHL-QS system: LuxI synthesizes endogenous AHL autoinducers that bind the cognate LuxR receptor, forming a LuxR–AHL complex that activates transcription of downstream target genes; in *P. plecoglossicida*, sRNA24 positively regulates this circuit by maintaining *luxR* mRNA steady-state levels. **(b)** LuxR solo responding to internal signals of other QS systems: The orphan LuxR receptor PplR recognizes heterologous AHL signals from cohabiting bacteria, coordinately regulating target gene expression alongside the canonical QS circuit. **(c)** LuxR solo independently responding to external signals: PplR directly senses exogenous AHL molecules without a cognate LuxI, independently modulating target gene transcription. These three modes collaboratively regulate interbacterial competition, host inflammatory responses, and biofilm formation in *P. plecoglossicida*.

The regulation of virulence factors also depends on cell density, with changes in population density detected through the autoinducers of QS systems (Azimi et al., 2020). In Gram-negative bacteria, LuxI/LuxR-type quorum sensing systems that employ N-acyl homoserine lactone (AHL) as signal molecules have been the most extensively studied (**Figure 5**) (Tang et al., 2019a; Yu et al., 2019). LuxI, acting as the signal synthase, catalyzes the production of AHL, which, upon binding by the transcriptional regulator LuxR, regulates the expression of genes associated with diverse phenotypes (Azimi et al., 2020). For many years, QS systems have been regarded as potential “antivirulence” targets, as they regulate the production of multiple virulence factors and host immune responses (Zhao et al., 2023). LuxR attenuates the pro-inflammatory immune response by suppressing the host NLR-IL-1β signaling axis, thereby promoting bacterial immune evasion and splenic granuloma formation. Downregulation of *luxR* expression significantly reduces the pathogenicity of this bacterium toward orange-spotted grouper, confirming that the LuxI/LuxR system is an important positive regulator of virulence in *P. plecoglossicida* (Zhao et al., 2023). Notably, among sequenced bacteria, more than 75% of LuxR-type receptors lack a paired LuxI-type autoinducer synthase, and such receptors are termed LuxR solo receptors (LuxR-solos) (Hudaiberdiev et al., 2015).

The LuxR solo protein PplR of *P. plecoglossicida* represents a key node that expands the boundaries of the classical quorum sensing network. Unlike classical LuxR receptors that are genomically adjacent to and paired with LuxI, PplR lacks endogenous AHL synthesis capability but retains the ligand-binding domain that recognizes exogenous AHL signals in the environment, thereby endowing this bacterium with the molecular basis for “signal eavesdropping” within complex microbial communities (Li et al., 2024). Studies have demonstrated that exogenous AHL extracts from *P. aeruginosa* significantly inhibit biofilm formation in wild-type *P. plecoglossicida* through PplR, whereas this response is abolished in the *pplR* deletion mutant, confirming PplR as a functional receptor mediating interspecies AHL signal transduction (Li et al., 2024). From a regulatory logic perspective, the PplR-AHL complex does not simply mimic the virulence-activating function of classical LuxR; instead, it drives the transition of the bacterium from a sessile to a planktonic lifestyle by negatively regulating the expression of biofilm matrix genes (Li et al., 2024). This strategy carries important ecological adaptive significance in multi-species aquatic environments or host lesion microenvironments: when the density of neighboring AHL-producing bacteria rises, *P. plecoglossicida*, via PplR, preemptively perceives the spatial occupation signals of competing flora and actively disperses to seek new colonization sites, thereby gaining an advantage in interspecies competition.

Moreover, quorum sensing information can be integrated and fine-tuned at the post-transcriptional level through sRNAs. In a recent study, *P. plecoglossicida* sRNA0024 was shown to positively maintain the signal output strength of this pathway by influencing *luxR* transcript levels. Deletion of sRNA0024 significantly reduced the mRNA abundance of *luxR*, and quorum sensing-associated virulence outputs (biofilm formation, host adhesion, and pathogenicity) were concomitantly attenuated (Zhao et al., 2025). This mechanism is highly similar to the mode of action of the Hfq-dependent sRNA00002 in *Pseudoalteromonas* sp. R3, which positively regulates the QS system by base-pairing with the 5′ UTR of *yasR* mRNA (encoding the LuxR-type AHL receptor) and enhancing its transcript stability (Mukherjee and Bassler, 2019; Wu et al., 2021). Thus, with LuxR as the core, PplR as the environmental sensing node, and sRNA0024 as the post-transcriptional tuning element, *P. plecoglossicida* has constructed a multi-layered QS network that enables it to dynamically calibrate its lifestyle and pathogenic strategies within complex microbial communities.

### 3.4 c-di-GMP

c-di-GMP is a key second messenger governing lifestyle transitions in bacteria. Its intracellular level is precisely controlled by two classes of functionally antagonistic enzymes: diguanylate cyclases (DGCs) containing GGDEF domains are responsible for its synthesis, whereas phosphodiesterases (PDEs) containing EAL or HD-GYP domains mediate its hydrolysis (Hengge, 2009; Römling et al., 2013). The genome of *P. plecoglossicida* encodes 29 GGDEF domain-containing proteins, 24 EAL domain-containing proteins, 15 GGDEF-EAL dual-domain proteins, and one HD-GYP protein, constituting a complex metabolic regulatory network (Wang et al., 2023). At the functional level, c-di-GMP mediates diverse regulatory effects through interactions with a variety of effectors, and its intrinsic structural flexibility further expands this functional diversity. Studies have shown that low c-di-GMP levels promote motility and the planktonic lifestyle, whereas high levels drive biofilm formation and sessile colonization (Bridges et al., 2022). However, the regulation of c-di-GMP in *P. plecoglossicida* exhibits more refined dose-dependent characteristics: its concentration must be maintained within an optimal window, as either excessively low or high levels result in virulence defects (Xu et al., 2016; Luo et al., 2019b).

YfiR, the negative regulator of the YfiBNR module, limits c-di-GMP synthesis by inhibiting the inner membrane DGC protein YfiN (Xu et al., 2016). Deletion of *yfiR* relieves this inhibition, significantly elevating intracellular c-di-GMP levels, driving the upregulation of biofilm matrix genes such as *alg8*, *algD*, and *bcs*, transforming the cells from a smooth, dispersed state into dense aggregates, while substantially impairing swimming and swarming motility (Wang et al., 2023). This lifestyle transition occurs at the cost of motility, rendering *P. plecoglossicida* unable to disseminate effectively from the initial infection foci and ultimately leading to markedly attenuated pathogenicity in the zebrafish larvae model. In contrast, the DGC gene L321_RS15240 is specifically highly expressed at the pathogenic temperature (18 °C) and rapidly reaches its expression peak following infection (Luo et al., 2019b). Silencing of this gene significantly reduces pathogenicity in orange-spotted grouper, with differentially expressed genes enriched in metabolic pathways (*prpC*, *ccoP-I*) and virulence-associated genes (*hemC*, *glpD*), suggesting that L321_RS15240 supports early colonization and survival of the bacterium in host tissues by coordinating metabolic adaptation and virulence gene expression (Luo et al., 2019b).

### 3.5 sRNAs

Bacterial sRNAs are a class of non-coding RNA molecules of 50–500 nucleotides in length that achieve rapid and precise regulation of gene expression at the post-transcriptional level through base pairing with target mRNAs (Jia et al., 2024; Khaledi et al., 2024). Their regulatory mechanisms exhibit marked bidirectionality: in negative regulation, sRNAs cause mRNA degradation or translational arrest by masking the ribosome binding site (RBS) of target mRNAs or by recruiting nucleases such as RNase E (Khaledi et al., 2024); in positive regulation, sRNAs can bind to the 5’ UTR of target mRNAs and remodel their secondary structures, exposing the shielded RBS and thereby promoting translation initiation and stabilizing the transcripts (Khaledi et al., 2024). The activity of sRNAs generally relies on mediation by RNA chaperone proteins (such as Hfq, ProQ, and CsrA), which promote and stabilize sRNA-mRNA pairing to enhance regulatory efficiency (Alquethamy et al., 2025).

In Gram-negative bacteria, sRNAs are extensively involved in multiple key physiological and pathogenic processes, including carbon metabolism, envelope stress responses, iron homeostasis, quorum sensing signal modulation, flagellar biogenesis, and virulence factor expression (Papenfort and Melamed, 2023). Notably, a single sRNA typically possesses the capacity to regulate multiple target mRNAs (McQuail et al., 2024). This “one-to-many” regulatory property enables sRNAs to act as central nodes in gene regulatory networks, synchronously coordinating the outputs of multiple functional pathways and linking and integrating two or more simultaneously active regulatory networks (McQuail et al., 2024).

In *P. plecoglossicida*, sRNAs likewise constitute an important component of the virulence regulatory network. Existing studies have identified several sRNA molecules with defined regulatory functions. sRNA0024 positively maintains the expression of the quorum sensing hub factor LuxR at the post-transcriptional level, thereby strengthening quorum sensing signal output (Zhao et al., 2025); sRNA113 and sRNA562 target the type III secretion system of the lateral flagella, negatively constraining bacterial motility and invasive capacity (He et al., 2024a, 2025b). Compared with the hundreds of sRNAs that have been systematically identified in model organisms such as *P. aeruginosa*, the number of functionally characterized sRNAs in *P. plecoglossicida* remains limited. The three functional sRNAs revealed to date are anchored in two core virulence pathways—quorum sensing and flagellar motility—suggesting that sRNAs in this bacterium may undertake extensive pathway integration and signal transduction functions, with additional virulence regulatory nodes yet to be identified.

## 4 Temperature-dependent virulence

Temperature is one of the fundamental environmental parameters affecting bacterial life processes (Moon et al., 2023). For pathogens, the abrupt temperature shift encountered upon entering or leaving a host is a key signal that initiates the infection program, inducing profound reprogramming of gene expression and metabolism, with virulence genes being the central regulatory targets of this reprogramming (Twittenhoff et al., 2020; Roncarati et al., 2025). Specifically, mammalian pathogens typically induce virulence genes at 37 °C (the host body temperature, which equals the optimal growth temperature); for example, the secreted toxin CnfY of *Yersinia pseudotuberculosis* is produced only at 37 °C (Twittenhoff et al., 2020). In contrast, the virulence genes of many fish pathogens, such as *Yersinia ruckeri*, *A. hydrophila*, and *E. tarda*, are markedly activated at temperatures below the optimal growth temperature (TBO) (Guijarro et al., 2015). Temperature-dependent virulence regulation is likewise widespread within the genus *Pseudomonas*, with patterns varying according to host niche. In the mammalian pathogen *P. aeruginosa*, virulence genes are activated at 37 °C (Wurtzel et al., 2012; Grosso-Becerra et al., 2014). In the plant pathogen *Pseudomonas syringae*, synthesis of the phytotoxin coronatine (COR) peaks below the optimal growth temperature and is mediated by the CorSRP two-component system (Smirnova et al., 2001, 2002), although strain-to-strain variation exists (Weingart et al., 2004). In the biocontrol strain *Pseudomonas fluorescens* CHA0, the synthesis of antifungal secondary metabolites (antibiotic compounds and hydrogen cyanide) is also temperature-dependent, being significantly higher at 30 °C than at 35 °C (Humair et al., 2009). Similarly, outbreaks of infection caused by the fish pathogen *Pseudomonas anguilliseptica*, a member of the same genus, are concentrated in low-temperature seasons (Doménech et al., 1997).

*P. plecoglossicida* is a typical representative of the TBO pattern within this genus: infection outbreaks occur mainly at water temperatures of 15 to 20 °C, whereas its optimal growth temperature is approximately 28 °C (Tao et al., 2016; Huang et al., 2018). Multi-omics analyses have revealed that *P. plecoglossicida* displays a distinct gene expression profile at the pathogenic temperature of 18 °C. Compared with non-pathogenic temperatures (12 °C and 28 °C), the pyoverdine (PVD) biosynthesis gene cluster (PVDS1 to 5) and T6SS core genes (*hcp*, *icmF*, and *dotU*) are specifically highly expressed at 18 °C (Huang et al., 2018). PVD siderophore activity is significantly higher at 18 °C than at 12 °C and 28 °C, and silencing of PVD biosynthesis genes markedly reduces iron acquisition capacity and pathogenicity (Huang et al., 2018). Given the critical role of iron-chelating systems in pathogenesis, it can be inferred that PVD is a key temperature-dependent virulence factor of *P. plecoglossicida*. Likewise, the T6SS is significantly activated at the pathogenic temperature. Hcp, the hallmark secreted protein of the T6SS, is markedly induced at 20 °C (Hcp2 being almost undetectable at 30 °C), and the transcription levels of T6SS genes are upregulated 2.5- to 4.2-fold relative to those at 30 °C (Tao et al., 2016). Silencing of T6SS genes (*hcp*, *dotU*, and *icmF*) impairs biofilm formation, host tissue colonization, and *in vivo* pathogenicity (Huang et al., 2018), confirming that the T6SS functions as a temperature-regulated effector injection apparatus that plays a critical role in host-pathogen interactions. Notably, Ding et al. (2025) found that T6SS-1 expression is driven primarily by monovalent cation signals such as Na^+^ and K^+^ sensed by the PvgAS two-component system, with temperature playing only a secondary, synergistic role.

In addition, multiple temperature-sensing regulatory systems cooperatively control the virulence output of *P. plecoglossicida*. Within the ECF σ factor family, RpoE and SigX are two core envelope stress-responsive regulators that assume distinct but complementary functional roles. SigX serves as a low-temperature-sensitive initiator of acute pathogenicity; its homolog in *P. aeruginosa* has been shown to respond to alterations in membrane physical properties rather than directly sensing temperature (Bouffartigues et al., 2020). In *P. plecoglossicida*, SigX is specifically highly expressed at the pathogenic temperature, and *sigX* deletion attenuates pathogenicity in the host and significantly remodels the serine-type endopeptidase, chemokine, and complement system pathways (Sun et al., 2018). This indicates that SigX likewise undertakes the key function of integrating low-temperature signals and initiating acute virulence in *P. plecoglossicida*, although whether its upstream sensing mechanism similarly involves alterations in membrane architecture awaits experimental validation. RpoE (an AlgU homolog), in contrast, acts as a broad-spectrum hub for envelope stress adaptation and T6SS regulation, with functions biased toward broad environmental adaptation and the maintenance of persistent infection: AlgU is a key σ factor involved in envelope stress and heat shock responses in *P. aeruginosa* (Bouffartigues et al., 2020), whereas *P. plecoglossicida* RpoE, although more strongly expressed at 28 °C, continuously and positively regulates T6SS-2 gene expression, interspecies competition, and macrophage invasion through the RIP cascade (Zhang et al., 2022). By competing for the RNA polymerase core enzyme, these two ECF σ factors form a complex temperature-virulence response network that enables *P. plecoglossicida* to achieve precise spatiotemporal control of gene expression at different temperatures.

Moreover, *P. plecoglossicida* relies on heat shock proteins and cold shock proteins to counteract the damage caused by temperature fluctuations and protect the cells. HtpG (a member of the HSP90 family) and CspA1 (a cold shock protein) are both induced and highly expressed at the pathogenic temperature, covering different dimensions of temperature stress, respectively. Silencing of *htpG* significantly reduces motility, biofilm formation, and tissue bacterial loads (Huang et al., 2019b); silencing of *cspA1* reduces biofilm formation by 4.11-fold and sharply increases the LD_50_ (Qi et al., 2019). This dual stress response elicited by the pathogenic temperature alters the conformational equilibrium of key regulatory proteins and the physical properties of membranes (Giuliodori et al., 2023). Together with the ECF σ factors, these proteins constitute the multi-layered temperature-virulence response network of *P. plecoglossicida*.

## 5 Conclusion and future perspectives

As an aquaculture pathogen with a broad ecological niche, *P. plecoglossicida* has evolved a sophisticated virulence system and regulatory network that underpin its dual adaptation to environmental survival and pathogenesis. This review has summarized current research progress on the virulence mechanisms of *P. plecoglossicida*. During long-term interaction and competition with its hosts, virulence factors are expressed in an efficient and orderly coordinated manner, supporting the bacterium in completing the entire infection cascade and conferring advantages in host colonization and niche competition.

Although remarkable progress has been made in understanding the virulence factors, regulatory networks, and temperature-dependent pathogenicity of *P. plecoglossicida*, many key scientific questions remain to be addressed: the direct interaction mechanisms between sRNAs and their target mRNAs, as well as the mediating functions of RNA chaperone proteins, await experimental validation; the cross-talk among regulatory layers such as QS, c-di-GMP, TCSs, and sRNAs remains unclear; and the coupling mechanisms between environmental factors (e.g., temperature and salinity) and the virulence regulatory network, together with the ways in which this bacterium dynamically adjusts its virulence strategies within different host microenvironments, require further elucidation.

Meanwhile, existing findings are outlining a clear translational path for disease control: the determinants summarized above constitute a portfolio of candidate antivirulence targets, including the T3SS master regulator ExsA (Zhang et al., 2017a, 2017b), the T6SS-1 structural components ClpV and Hcp (Luo et al., 2019a; Jin et al., 2021; Zeng et al., 2022), the PvgAS ion-temperature switch (Ding et al., 2025), and the LuxR solo receptor PplR (Li et al., 2024); and a series of live attenuated and subunit vaccine candidates have shown promising protection in large yellow croaker (Ye et al., 2021; Li et al., 2022a; He et al., 2023a, 2024; Kang et al., 2024; Ahat et al., 2026; Li et al., 2026), highlighting the feasibility of translating mechanistic insights into applications. Admittedly, the genetic stability, biosafety, and field applicability of these candidates still await systematic evaluation. With continued mechanistic dissection and the accumulation of candidate targets and vaccine strains, advancing them toward safety assessment and aquaculture practice will remain an important direction for future research and translational application.
